# The Organoid Decade: Leveraging 3D Patient-Derived Organoids to Bridge the Translational Gap in Triple-Negative Breast Cancer: A Systematic Review

**DOI:** 10.3390/cells15100922

**Published:** 2026-05-18

**Authors:** Jemima Sani, Bin Yi, Yaguang Xi

**Affiliations:** 1 Department of Pharmaceutical and Biomedical Sciences, College of Pharmacy, University of Georgia, Athens, GA 30602, USA; jss02782@uga.edu (J.S.); bin.yi@uga.edu (B.Y.); 2 Innovations in Drug Discovery (IDD) Program, College of Pharmacy, University of Georgia, Athens, GA 30602, USA

**Keywords:** immunotherapy, T cell exhaustion, tumor heterogeneity, tumor-associated macrophages, tumor microenvironment, artificial intelligence, precision medicine, regulatory intelligence

## Abstract

Triple-negative breast cancer (TNBC) is an aggressive subtype of breast cancer with limited effective therapies. Two-dimensional (2D) *in vitro* models poorly recapitulate tumor microenvironment (TME) interactions, impeding the translational relevance of TNBC immunotherapy research. Three-dimensional patient-derived tumor organoids (3D PDTOs) have emerged as advanced preclinical models that better mimic tumor–immune interactions. The objective of this systematic review was to assess the landscape of 3D PDTO adoption in TNBC research and evaluate their application in addressing key bottlenecks in TNBC immunotherapy. We retrieved 394 studies published between 2015 and 2025 from the PubMed and ClinicalTrials.gov databases. Of those, 153 studies were included in the review. Fifty-eight (58) TNBC-specific studies met the inclusion criteria, including explicit mention of 3D PDTOs in the title or abstract, with confirmation in the Methods and Results sections. Studies were excluded if they used non-patient-derived tumor organoids or referred to other 3D models as 3D PDTOs. Data were collected from January 2025 through December 2025. Eligible studies were screened in three (3) tiers, grouped by relevant themes and graphed in Excel. We present an overview of the adoption of 3D PDTOs in TNBC research, highlighting the most common application trends within this scope. We also discuss the potential impact of artificial intelligence (AI) and regulatory guidance from the United States Food and Drug Administration (FDA) and the European Medicines Agency (EMA) pertinent to the adoption of organoids as human-relevant models to improve translational outcomes. Overall, this review provides actionable insights for leveraging 3D PDTOs to advance translational TNBC research and precision oncology.

## 1. Introduction

Triple-negative breast cancer (TNBC) is a heterogeneous subtype of breast cancer that lacks the three key receptors found in other breast cancer subtypes: estrogen receptor (ER), progesterone receptor (PR), and human epidermal growth factor receptor 2 (HER2) [1,2]. TNBC is widely recognized as one of the most aggressive breast cancer subtypes and is associated with poor prognosis and reduced overall survival.

Heterogeneity has been identified as a key factor limiting the development of effective therapeutic options for patients with TNBC. Specifically, heterogeneity among tumor components and immune cells within the tumor microenvironment has been cited as a major factor contributing to treatment failure and resistance [3,4]. Recent advances in immunotherapy have expanded the TNBC treatment landscape by providing an additional therapeutic option. In 2020, the U.S. FDA granted accelerated approval for pembrolizumab, an anti-programmed cell death protein-1 antibody (anti-PD-1) immunotherapy. It was approved to be used in combination with chemotherapy for patients with PD-L1-positive locally recurrent, unresectable or metastatic TNBC. The approval was based on the increase in progression-free survival (PFS) to 9.7 months (95% CI: 7.6–11.3) compared to the 5.6 months (95% CI: 5.3–7.5) in the placebo arm [5].

Less than a year later, pembrolizumab was also approved for high-risk early-stage TNBC based on an increased pathological complete response (pCR) compared to chemotherapy alone [6]. However, the efficacy, including objective response rates and durability of benefit, remains largely confined to a subset of patients, even within PD-L1-positive disease. In addition, clinical experience and translational studies have increasingly described acquired resistance after an initial response, underscoring that immune escape can emerge during therapy and limit long-term disease control in many patients.

The complex composition of the tumor microenvironment (TME) has been shown in several studies to influence immunotherapy outcomes across cancer subtypes, even beyond TNBC [7,8]. Concerns about the limited ability of traditional two-dimensional (2D) cell models used in non-clinical research to recapitulate heterogeneity have emerged as a major pain point in the scientific community. In particular, the inability of these 2D models to accurately mimic cellular and molecular interactions within the TME may limit their predictive value for immunotherapy responses [9,10,11]. Overall, 90% of therapies deemed efficacious in non-clinical research fail to translate into safe and effective clinical responses [12]. This translational gap highlights the urgent need for the adoption of more predictive, human-relevant models in non-clinical research.

Three-dimensional patient-derived tumor organoids (3D PDTOs), defined as miniaturized, clone-like structures that recapitulate the key features of the originating patient’s tumor, have emerged as promising human-relevant models to bridge the translational gap in immuno-oncological research. 3D PDTOs are pivotal in faithfully recapitulating critical interactions within the TME [13]. The versatility of 3D PDTOs offers diverse opportunities to leverage them for modeling heterogeneity and tumor–immune interactions, two key bottlenecks in TNBC immuno-oncology research. Compared to animal models, 3D PDTOs are more time- and cost-efficient for non-clinical studies with the added advantage that they can also be leveraged for clinical research [14,15,16].

Despite the strong potential of 3D PDTOs to recapitulate key aspects of *in vivo* tumor progression within the original patient-specific TME in a way previously impossible, routine adoption of the technology in conventional research and clinical applications still lags [13]. The lack of a standardized protocol, together with labor-intensive workflows, substantial time demands, and high costs, has been widely cited as a major barrier to the broader adoption of 3D PDTOs [10].

**Figure 1:**
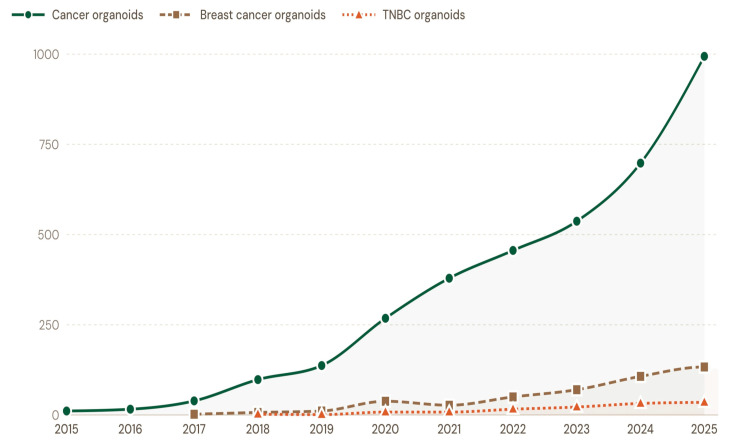
Publication trends on patient-derived cancer organoids, breast cancer organoids, and TNBC organoids indexed in PubMed from 2015 to 2025. Annual publication counts were generated using predefined PubMed search strings. The data show a substantial increase in patient-derived cancer organoid literature over time. Publications focused on breast cancer organoids and TNBC organoids also increased steadily, although they remained smaller subsets of the broader patient-derived organoid research landscape.

**Figure 2:**
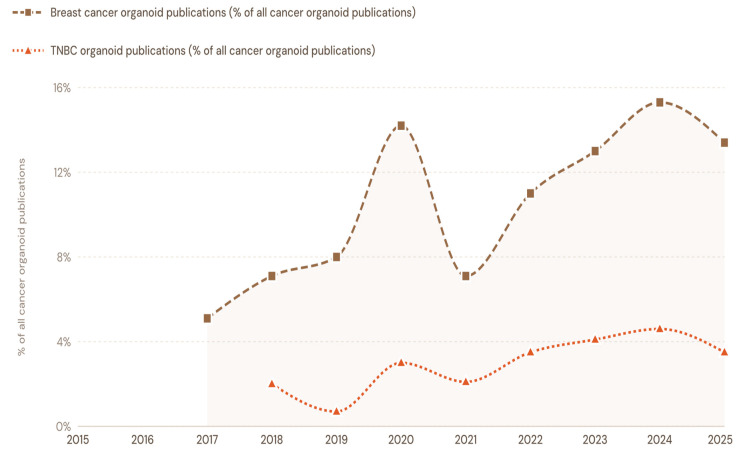
Annual proportion of PubMed-indexed patient-derived cancer organoid publications focused on breast cancer and TNBC. Breast cancer organoid studies accounted for a variable but generally increasing proportion of the patient-derived cancer organoid literature over time. TNBC organoid studies also increased proportionally, although they remained a smaller subset of both the breast cancer organoid and broader patient-derived cancer organoid publication landscape.

From 2015 to 2025, PubMed indexed 3633 publications on patient-derived cancer organoids, including 480 on patient-derived breast cancer organoids and 124 on patient-derived TNBC organoids. While all categories showed an upward trend across the decade, adoption of 3D PDTOs in TNBC research remained comparatively limited, reflecting steady growth but continued underrepresentation relative to the broader breast cancer and overall cancer organoid literature. Figure 1 and Figure 2 highlight the trends of TNBC organoid adoption relative to overall breast cancer organoid adoption and patient-derived cancer organoid studies indexed in the PubMed database.

The primary objectives of this systematic review are to critically examine the trends in the adoption of 3D PDTOs in non-clinical and clinical TNBC research over the past decade; assess how 3D PDTOs are being leveraged to address common immunotherapy bottlenecks in TNBC; evaluate the regulatory guidelines governing the adoption of the human-relevant 3D models in the United States and the European Union (EU); and highlight the impact of artificial intelligence on TNBC studies involving 3D PDTOs.

The outcomes of interest are the most common methods, applications, and limitations associated with the adoption of 3D PDTOs in TNBC research, the specific applications of 3D PDTOs in investigating T cell exhaustion, macrophage dysregulation, and tumor heterogeneity in TNBC studies; a summary of regulatory milestones and official communication pertinent to 3D PDTOs; and an assessment of the impact of artificial intelligence (AI). The summaries are presented as tables and graphs in-text and in the Supplementary Materials.

Overall, we summarize state-of-the-art methodologies and experimental approaches used in TNBC organoid research and discuss key challenges and persistent gaps that must be addressed to enable the effective integration of 3D PDTOs into routine TNBC research.

## 2. Materials and Methods

We systematically searched PubMed for published studies and ClinicalTrials.gov for registered studies related to triple-negative breast cancer and patient-derived tumor organoids. Using targeted keywords and Boolean operators, we applied search strings such as “triple-negative breast cancer” AND “organoids” AND “patient derived triple-negative breast cancer organoids” AND “T cell exhaustion” along with additional related combinations, as presented in the Supplementary Materials.

Records retrieved from each source were exported for deduplication and screening. After eligible studies were selected, they were organized into eight thematic categories for analysis: organoid-focused, immunotherapy-focused, tumor microenvironment-focused, precision oncology-focused, triple-negative breast cancer-focused, T cell exhaustion in TNBC-focused, T cell exhaustion in organoids-focused, and alternative search. These themes were used only to classify the included studies during analysis and were not incorporated into the Boolean database search strategy.

From ClinicalTrials.gov, we retrieved 203 studies. Additionally, we collected organoid-relevant publications from the FDA and EMA official websites, and cancer statistics studies from the American Cancer Society (ACS) website and other alternative searches. The review followed an unregistered internal protocol for searching, screening, eligibility assessment, and thematic subcategorization. The protocol summary is provided in the Supplementary Materials.

For the TNBC-specific analyses, only studies confirmed to leverage 3D PDTOs were included. The predefined inclusion criteria for TNBC-specific studies included in the analysis were: TNBC studies published between 2015 and 2025 that reported the use of 3D PDTOs in the titles or abstracts, with confirmation in the Methods and Results sections. The exclusion criteria were studies using non-patient-derived organoids; studies that report the use of spheroids or other 3D models as organoids; preprints; and non-English or untranslated publications. Studies that met the inclusion criteria were exported to an Excel sheet from January 2025 to December 2025. Duplicate records were manually excluded during the data entry into the Excel sheet, and EndNote (v2025.2) was used as a citation management tool to detect and merge any potential duplicate citation. Following deduplication, the final reference list was reviewed for further validation.

A three-tier review approach was conducted by an individual investigator to confirm the specific reporting, models, and application of 3D PDTOs across the included studies (titles, abstracts, and Methods sections). This screening was repeated three times. The full-text articles were then assessed with the same inclusion and exclusion criteria. A second investigator subsequently and independently reviewed the analysis output to verify reporting consistency, and disagreements were resolved by discussion.

Methodological quality was assessed using study design-appropriate appraisal tools, as described below. We used a three-step verification process to reduce misclassification and reporting bias in the TNBC-specific studies included in the analyses. We also sought to identify and minimize other sources of bias by reporting database selection, search strings, search period, predefined inclusion and exclusion criteria, and practical time and scope constraints in line with the Preferred Reporting Items for Systematic Reviews and Meta-Analyses (PRISMA) guidance. When primary studies did not directly report the outcomes of interest, we summarized and/or cited relevant reviews published within the predefined timeframe, which may introduce additional bias. Accordingly, the findings should be interpreted as descriptive and are limited to the prespecified criteria.

To assess the translational landscape, we reviewed relevant regulatory guidance documents from the FDA and EMA. Overall, the data collection and reporting followed the PRISMA guidelines for systematic reviews. PRISMA is a widely used reporting framework that promotes transparency and reproducibility by specifying the key information that should be documented, including the search strategy, study selection process, eligibility criteria, and synthesis of results, often summarized in a flow diagram.

## 3. Results

**Figure 3:**
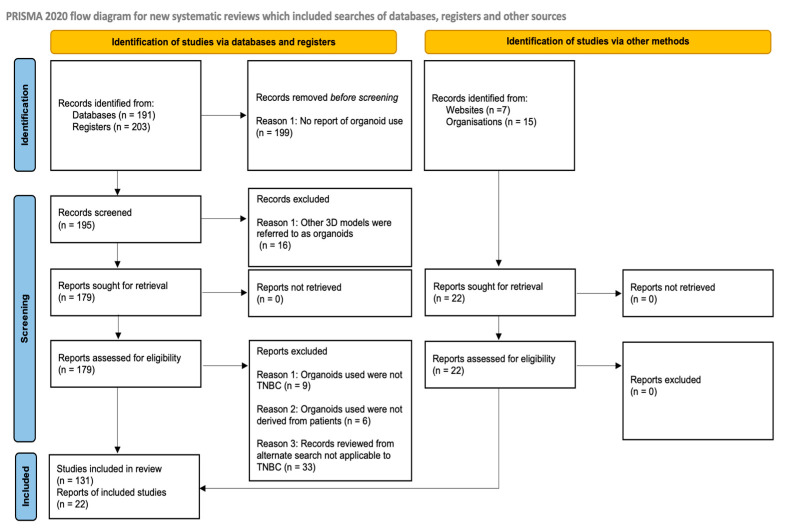
The PRISMA 2020 flow diagram illustrates the stages of study identification, screening, eligibility assessment, and inclusion for this systematic review. Arrows indicate progression through identification, screening, eligibility, and inclusion.

Figure 3 shows the PRISMA 2020 flow diagram for literature identification and screening.

### 3.1. TNBC Classification

One of the first published subclassifications of TNBC was reported by Lehmann et al. in 2011 [17]. Using gene expression profiling of tumor samples from 587 patients, they classified TNBC into six distinct subtypes: basal-like 1 (BL1), basal-like 2 (BL2), mesenchymal (M), mesenchymal stem-like (MSL), immunomodulatory (IM), and luminal androgen receptor (LAR) [17]. In 2015, Burstein et al. investigated 198 previously uncharacterized TNBCs using mRNA expression and DNA profiling. They identified four distinct TNBC subtypes: luminal/androgen receptor (LAR), mesenchymal (MES), basal-like immunosuppressed (BLIS), and basal-like immune-activated (BLIA) [18]. Although the classification by Burstein et al. identified basal-like subtypes broadly consistent with the immunosuppressed and immune-activated TNBC groups, it did not recapitulate the distinct IM and MSL subtypes reported by Lehmann et al.

The following year, Lehmann et al. reanalyzed data from five public gene expression datasets [19]. They redefined TNBC from six to four subtypes: BL1, BL2, M, and LAR. The team found that the characteristics of the IM and MSL subtypes were due to tumor infiltrating lymphocytes (TILs) and tumor-associated stromal cells, as determined by gene expression and pathological analyses [19].

Since then, several other groups have published various subclassifications of the disease based on different factors, including molecular, morphological, and clinical features [20,21,22]. The standard clinical classification of TNBC remains limited to immunohistochemistry (IHC), with genetic testing increasingly incorporated to improve diagnostic and therapeutic stratification [23,24]. Overall, classification trends still retain the umbrella structure, grouping all subtypes under a single group. This reflects gaps and inefficiencies in the current classification and diagnosis of TNBC. To ensure timely and accurate TNBC diagnosis in patients, TNBC needs to be better characterized during diagnosis to determine the best treatment option based on specific subtypes.

Today, human-centered research is advancing beyond immunohistology, transcriptomics, and morphological classification. In particular, advances in 3D PDTO technology may enable more precise identification and modeling of TNBC subtypes that were previously difficult to classify using conventional methods [25].

### 3.2. 3D TNBC PDTOs

3D PDTOs are microstructures established from primary patient tumor samples. They are cultured *in vitro* with specialized media containing a cocktail of growth factors that facilitate cell self-organization and modeling of the originating tumor. In addition to tumor cells, organoids typically retain immune and stromal cells from the patient [26]. The ability of 3D PDTOs to recapitulate the TME makes them an invaluable resource for TNBC research. Our review of the historical development of 3D PDTOs in TNBC research found that the current 3D culture model used to establish PDTOs across laboratories today stems from the technique developed by Wilson in 1907 [27]. Wilson reported that, under suitable conditions, dissociated sponge cells could reaggregate into small masses of undifferentiated tissue, which could subsequently grow and differentiate into complete sponges. He also revealed how critical microscopic analysis over several weeks was pivotal in uncovering a series of morphological hallmarks involved in the successful regeneration of *in vitro* organisms [27,28].

In 1987, Zimmermann reported the successful development of lung organoid cultures from mouse fetuses. Zimmermann employed microscopic imaging to extensively characterize developmental changes and morphological events in lung organoid culture. The key morphological events revealed by microscopic images taken of the organoid culture over time included cell sorting, epithelial–mesenchymal interactions, formation of alveolar-like lumen, and basal lamina development. Zimmermann described the use of a medium–air interface culture technique to establish the organoid culture and highlighted the importance of fibroblast-derived factors, matrix components, and epithelial–mesenchymal interactions in facilitating the morphogenic development process. Zimmermann further used the established lung organoid cultures to screen a series of compounds including corticosteroids and retinoids, to assess their effects on organoid proliferation and growth inhibition [29].

In 2009, Sato et al. reported remarkable success in developing self-organizing 3D mouse intestinal crypt-villus structures from a single stem cell in the absence of a non-epithelial cellular niche. Sato et al. disclosed that the mechanism for establishing 3D organoids involves multiple crypt fission events and the simultaneous generation of villus-like epithelial domains containing all differentiated cell types [30]. By 2011, Sato et al. had adapted the culture conditions to grow similar epithelial organoids of the mouse colon, the human colon, and the human small intestine [31]. This advancement in biology has given rise to the modern-day culture of 3D patient-derived organoids.

One of the earliest reports of the successful establishment of 3D TNBC PDTOs was in 2018 [32]. Mazzucchelli et al. also reported successful establishment of patient-derived TNBC organoids in 2019 [33]. Four years later, Bhatia et al. established a diverse biobank of TNBC PDTOs and carried out a comprehensive analysis and characterization to validate their ability to recapitulate the patients’ originating tumors. The group identified a series of genomic alterations, transcriptomic signatures, cell-type specificity, and morphological characteristics, which validated the ability of 3D PDTOs to mimic their originating tumors [3]. Some of the applications that Bhatia et al. confirmed that TNBC PDTOs can be used for include drug screens, co-culture experiments, metabolomics, and fate-mapping studies to better understand the mechanisms driving cancers and identify more effective treatment options [3].

**Figure 4:**
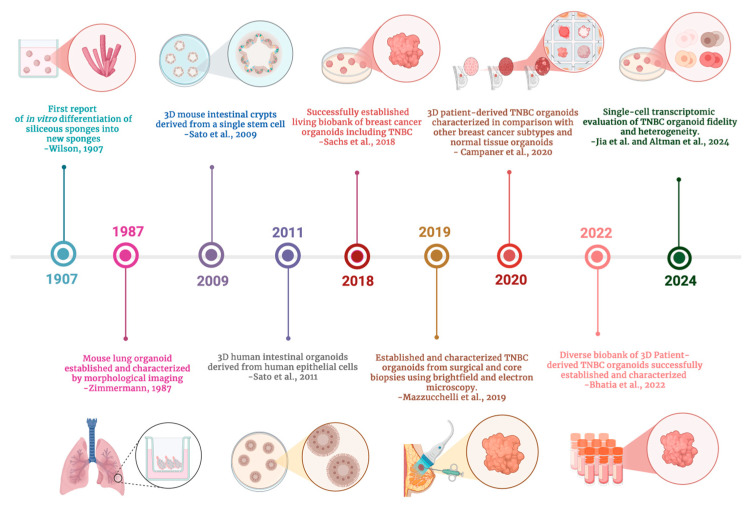
Timeline of key milestones in 3D and organoid culture leading to the successful establishment of 3D patient-derived TNBC organoids. Major advances include early observations of *in vitro* cellular self-organization by Wilson (1907) [27]; the establishment and characterization of mouse lung organoids by Zimmermann (1987) [29], the development of mouse and human intestinal organoids by Sato et al. (2009, 2011) [30,31]; and the establishment, characterization, biobanking, drug-response modeling and single-cell transcriptomic evaluation of 3D patient-derived TNBC organoids by Sachs et al. (2018), Mazzucchelli et al. (2019), Campaner et al. (2020), Bhatia et al. (2022), Jia et al. (2024) and Altman et al. (2024) [3,16,32,33,34,35]. (Created in https://BioRender.com).

Of note, in 2020, Campaner et al. characterized 3D PDTOs from multiple breast cancer patients including TNBC patients. Campaner et al. found 3D PDTOs to have histologic and genomic concordance to originating tumors. They noted that 3D PDTOs established from TNBC patients in their study were easier to identify as malignant. TNBC 3D PDTOs from the Campaner et al. study were distinctly characterized by large cells with solid growth patterns, appearance of prominent macronuclei and higher proliferation rates compared to other subtypes and normal-appearing adjacent-to-tumor specimens. However, they also noted that 3D PDTOs often contain diverse cell populations that may differ from those of the originating tumor. Despite this limitation, Campaner et al. reported 3D PDTOs as valuable tools to evaluate drug efficacy and resistance patterns within tumors [34]. Figure 4 shows the timeline of fundamental advancements that serve as pillars governing the adoption of 3D PDTOs in TNBC research across most laboratories in the last organoid decade.

Overall, these studies suggest that TNBC organoids can be efficiently established from patient-derived tumor samples. However, the successful establishment of 3D PDTOs varies substantially by tumor type, molecular subtype, specimen quality, and culture protocol. In TNBC, reported organoid establishment rates range from approximately 19% to 77%, whereas luminal and HER2-positive breast cancer subtypes have shown reported ranges of 20% to 96% and 41% to 92%, respectively [36]. Comparable variability is also observed across other cancer types. Metastatic colorectal cancer organoids are often established with relatively high efficiency, ranging from approximately 75% to 86% [37]. Pancreatic ductal adenocarcinoma organoid establishment rates vary more widely, ranging from approximately 16% to 91% [38]. In contrast, prostate cancer organoids remain comparatively difficult to establish, with overall success rates reported at approximately 15% to 20% [39].

To determine the extent to which 3D PDTOs preserve patient tumor biology, Bhatia et al. applied single-cell transcriptomics to compare cells from the original tumor samples with those from 3D organoid cultures. Bhatia et al. found that 3D PDTOs recapitulate the various subtypes and molecular signatures of breast biopsy samples. In non-malignant 3D mammary organoids, major cell types found in mammary epithelium are typically conserved. By contrast, 3D PDTOs derived from TNBC tumors lose mammary epithelial lineage specificity and are predominantly enriched in luminal progenitor-like cells [3].

Jia et al. investigated discrepancies in breast cancer organoid recapitulation using single-cell transcriptomic analysis. Similar to the findings of Bhatia et al., they conducted an extensive analysis of paired tumor and 3D PDTO breast cancer samples. The study included 66,920 quality-controlled cells for this robust analysis. Results from their study showed a significant reduction in immune and stromal cells in the 3D PDTOs compared with the primary tumors; however, epithelial cells were well conserved. They confirmed 3D PDTOs exhibit stronger and cleaner copy number variation signals, though retention patterns vary across molecular subtypes [35].

TNBC 3D PDTOs specifically enriched for claudin-low cells from the originating tumors, with increased MKI67 expression levels, indicating higher proliferation activity. TNBC 3D PDTOs also well preserved the migratory cell subgroup; however, the angiogenesis, stemness, and hypoxia subgroups were markedly reduced. This reduction limited the overall genomic concordance of TNBC 3D PDTOs by approximately 20% compared with other breast cancer subtypes [35].

It is known that HR-positive breast cancer subtypes preserve 88.2% of copy number variations, and TNBC preserves 62.4%. Jia et al. investigated this variation further and attributed this difference to the oxygen-enriched organoid culture conditions predominantly used in current TNBC organoid culture. Primary TNBC tumors have a partial pressure of oxygen of 10 mmHg, while organoid cultures usually have 150 mmHg. Jia et al. also found that TNBC 3D PDTOs grown under these standard organoid conditions were more sensitive to cisplatin. In contrast, other subtypes maintained the drug sensitivity seen in primary tumors [35].

Jia et al. found that while current organoid culture conditions suffice for other breast cancer subtypes, the loss of key features in TNBC 3D PDTOs that may be attributed to the culture conditions can compromise their utility for drug sensitivity assays. Based on the findings from their study, Jia et al. emphasized the need for enhanced quality control during TNBC 3D PDTO culture [35].

Altman et al. also highlighted the need for improved organoid culture conditions to enhance the accuracy of 3D PDTO recapitulation for drug-sensitivity assays and broader research and clinical applications. In their study examining transcriptional differences between patient-derived xenograft (PDX) and patient-derived xenograft organoid models, they found a significant difference in metabolic genes across the models. PDX organoids highly upregulated glutamine-cystine ligase catalytic and regulatory subunits, and several aldo-keto reductase family genes. The changes in metabolic genes suggest a potential shift in cellular metabolism towards altered redox regulation. A similar trend in altered cellular metabolism is typically observed in *in vitro* culture systems and may impact the interpretation of data obtained from 3D PDTOs [16].

A limitation stated in the Jia et al. study was that drug sensitivity analysis was determined solely through *in silico* analysis, and further *in vitro* validation is needed to confirm the observations. The Altman et al. study also only compared the difference in the organoid models with PDX tumors and not 3D PDTOs derived from primary tumor samples to primary tumor samples; thus, their findings need further validation in comparing 3D PDTOs to matching primary tumors and PDXO organoids to primary patient tumors. This is especially important because of the positive correlation observed in clinical applications, where 3D PDTOs accurately predicted patient responses.

### 3.3. 3D PDTOs in TNBC Research

**Figure 5:**
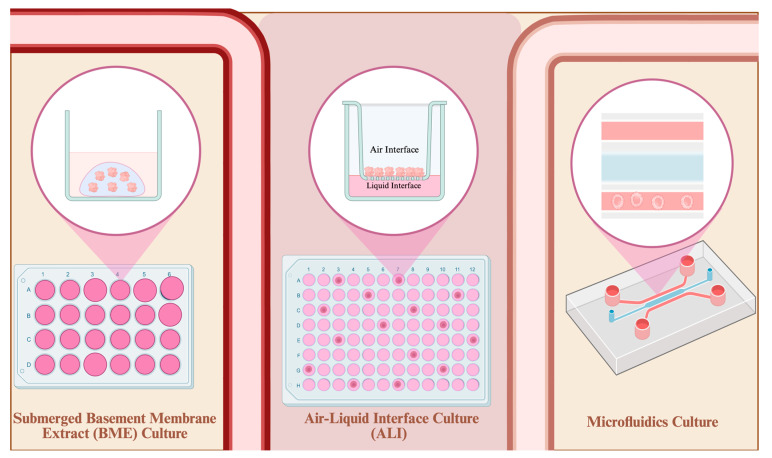
Three major methods used in establishing 3D PDTOs in TNBC research from 2015 to 2025. Submerged BME cultures embed patient-derived organoids within a basement membrane matrix to support 3D growth. ALI cultures expose the apical surface to air while supplying medium basolaterally, promoting epithelial differentiation and polarization. Microfluidic systems use chip-based channels to provide controlled flow and dynamic biochemical and mechanical cues, thereby increasing physiological relevance. (Created in https://BioRender.com).

To investigate the 3D PDTO models used in TNBC research, we screened the Methods sections of all studies meeting our inclusion criteria and verified the organoids used were established from primary TNBC patient tumor samples. We identified three major organoid models commonly employed in TNBC research from 2015 to 2025. These models are the submerged basement membrane extract (BME), air-liquid interface (ALI), and 3D microfluidic culture models. Figure 5 shows these three major methods used to establish 3D PDTOs in TNBC research.

#### 3.3.1. Submerged BME Culture Model

**Figure 6:**
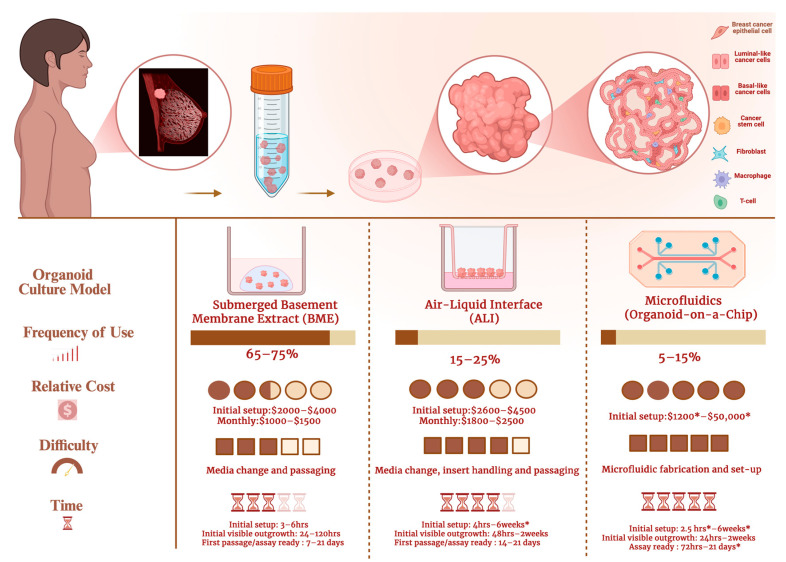
Comparison of major organoid culture models used in establishing 3D patient-derived organoid models in TNBC research in the 2015–2025 organoid decade. The upper panel illustrates the workflow from breast tumor sampling to tissue processing, organoid culture, organoid growth, and cellular-composition assessment. Brown arrows indicate workflow progression, and pink circular insets indicate magnified views. The color-coded cell legend identifies breast cancer epithelial cells, luminal-like cancer cells, basal-like cancer cells, cancer stem cells, fibroblasts, macrophages, and T cells. The lower panel compares submerged basement membrane extract (BME), air-liquid interface (ALI), and microfluidic organoid-on-a-chip culture systems. The bar icon indicates frequency of use, dollar icon relative cost, gauge icon technical difficulty, and hourglass icon time requirement. Dark brown filling represents the estimated level for each parameter, while pale yellow/beige represents the remaining scale; greater dark-brown filling indicates higher use, cost, difficulty, or time. Estimated use frequencies are 65–75% for submerged BME, 15–25% for ALI, and 5–15% for microfluidics. Estimated costs are $2000–$4000 initial setup and $1000–$1500/month for submerged BME; $2600–$4500 initial setup and $1800–$2500/month for ALI; and $1200*–$50,000* initial setup for microfluidics. Estimated timing ranges are 3–6 h setup, 24–120 h visible outgrowth, and 7–21 days to first passage/assay readiness for BME; 4 h–6 weeks* setup, 48 h–2 weeks visible outgrowth, and 14–21 days to first passage/assay readiness for ALI; and 2.5 h*–6 weeks* setup, 24 h–2 weeks visible outgrowth, and 72 h–21 days* to assay readiness for microfluidics. Asterisks indicate ranges that vary depending on system complexity, device design, fabrication needs, or laboratory workflow. Included studies are listed in Supplementary Materials [3,44,45,46,47,48,49,50,51,52,53,54,55,56,57,58]. (Created in https://BioRender.com).

The submerged BME culture involves suspending dissociated patient-derived TNBC tumor samples in a viscous hydrogel that simulates the extracellular matrix (ECM) environment around the tumor and TME. The hydrogel solidifies at temperatures between 22 °C and 37 °C. Once solidified, the hydrogel forms a jelly-like 3D dome, allowing the suspended cells from the patient’s tumor biopsy to differentiate into 3D structures. As shown in Figure 6, we found the submerged BME culture to be the most common method of culturing TNBC organoids. Applications of the submerged BME organoid culture from 2015 to 2025 include disease modeling, drug-response, and mechanism-of-action studies [40,41].

#### 3.3.2. ALI Culture Model

The ALI culture model involves culturing 3D PDTOs in the upper chamber of a transwell plate to increase oxygen exposure. This setup has been used to support long-term organoid culture, while also helping address oxygen-diffusion limitations that can contribute to hypoxia and necrosis in larger 3D organoids [26,42]. Despite the relevance of hypoxia to *in vivo* solid tumor biology, it is not exclusive to malignant organoids. Non-malignant 3D organoids are also affected by limited oxygen diffusion and absence of vascularization. Excessive hypoxia in larger 3D PDTOs can confound experimental results and severely compromise organoid viability, in some cases leading to sample loss [43].

ALI 3D PDTOs are also reported to be better suited for the retention and survival of patient tumor-intrinsic fibroblasts and immune cells [11,26]. This prolonged retention allows for the simulation and observation of native tumor–immune interactions to a considerable extent, reflecting interactions that occur within the originating patient TME. Over the decades, applications of ALI models in TNBC studies have primarily focused on drug testing of novel immunotherapeutic options and therapies [50,59]. The ALI technique is still underutilized for studying tumor–immune interactions in TNBC.

#### 3.3.3. 3D Microfluidics Culture Model (Organoid-on-a-Chip)

3D microfluidics platforms are minuscule culture compartments designed to keep the 3D PDTOs at micrometer-scale sizes [60]. The 3D microfluidics culture emerged as an alternative to the traditional 3D organoid culture methods discussed above. The development of 3D microfluidics aims to reduce the time and labor-intensive processes of scaling up 3D PDTOs [61,62]. Additional advantages of establishing smaller 3D TNBC PDTOs include reduced size variability and improved regulation of hypoxia, both of which may help address reproducibility and standardization challenges in organoid research [63]. Despite their smaller size, microfluidic 3D PDTOs can recapitulate key intercellular interactions within the originating patient tumor niche and have been validated for treatment-response prediction [8,60].

### 3.4. Applications of 3D PDTOs in Non-Clinical TNBC Research

**Figure 7:**
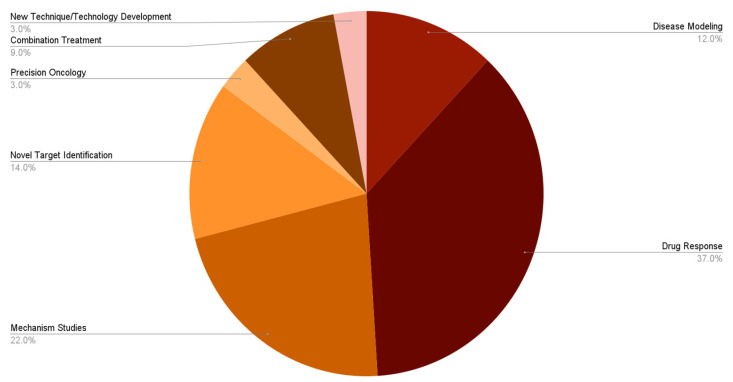
Trends of the applications of 3D PDTOs in TNBC studies published between 2015 and 2025. Drug response and therapeutic sensitivity testing represent the largest category (37.0%), followed by mechanistic studies (22.0%) and novel target identification (14.0%). Smaller proportions of studies focus on disease modeling (12.0%), combination treatment strategies (9%), and precision oncology applications (3.0%). Percentages were rounded to the nearest whole number to eliminate decimal values. Studies included are found in the Supplementary Materials [3,10,25,45,50,52,56,58,63,64,65,66,67,68,69,70,71,72,73,74,75,76,77,78,79,80,81,82,83,84,85,86].

We analyzed over fifty (50) publications from 2015 to 2025 that adopted 3D PDTOs in TNBC research. In this section, we present our findings and provide detailed insights into how 3D PDTOs are leveraged in non-clinical TNBC research. Figure 7 shows the percentage trends of the application of 3D PDTOs over the last decade. The major applications of 3D PDTOs in TNBC include drug response, disease modeling, and mechanism-of-action studies. Other applications include novel target identification, precision oncology, and the evaluation of new combination treatment efficacy.

#### 3.4.1. Drug Efficacy and Response

The use of 3D PDTOs in drug screening and treatment validation emerged as the most common application of the model in non-clinical TNBC research. From screening novel therapeutics and new combination treatment options to radiation therapies, the versatility of 3D PDTOs has enabled the discovery of novel, efficacious strategies that could be developed into more effective treatments for patients with TNBC.

Several studies included in our review used 3D PDTOs to evaluate drug responses in TNBC, including those by Ruan et al., He et al., and Rao et al., who applied these models to identify novel therapeutic strategies [56,58,87]. Rao et al. used 3D PDTOs to screen 169 epigenetic compounds and reported that organoid-based systems provided greater precision in drug response assessment than conventional cell-based models. Their study identified four compounds, panobinostat, pacritinib, TAK-901, and JIB-04, as potential epigenetic agents for TNBC treatment [87]. He et al. used these models to identify a novel AXL receptor tyrosine kinase degrader that primarily targets AXL, a kinase known to play important roles in TNBC tumorigenesis, metastasis, and drug resistance [56]. Ruan et al. also leveraged 3D PDTOs to support the discovery of iridium(III) pyridinium N-heterocyclic carbene complex 1 as a potential anti-tumor agent for TNBC and demonstrated the ability of 3D PDTOs to predict efficacy at low micromolar concentrations [58]. Furthermore, Ge et al. demonstrated the use of 3D PDTOs to uncover that the loss of tektin4 increases interactions between HDAC6 and α-tubulin, decreases microtubule stability, and sensitizes TNBC to ACY1215, a HDAC-selective inhibitor [88]. The application of 3D PDTOs in epigenetic studies highlights the potential of these models to be used as robust systems to model and interrogate unknown mechanisms of epigenetic modifications and answer long-sought knowledge gaps on how aberrant epigenetic modifications are implicated in TNBC tumorigenesis. The ability of 3D PDTOs to retain epigenetic changes across individual patients makes them preferable for personalized oncology over other 3D models. Emerging applications in HDAC-directed studies merit further studies to validate the application of 3D PDTOs in epigenetic TNBC research.

Zhao et al. employed 3D PDTO models to discover novel combination treatment options against TNBC and found that the combination of chidamide and enzalutamide had a synergistic effect, thereby improving treatment efficacy across TNBC subtypes [50]. Chew et al. evaluated the effects of FGFR in breast cancers using 3D PDTOs and phosphoproteomic profiling across 18 TNBC PDX models. By integrating multi-omics into these models, they identified novel fibroblast growth factor receptors (FGFR) fusions that would otherwise have gone undetected by whole-exome sequencing. They also evaluated the efficacy of selective tyrosine kinase inhibitors AZD4547 (FGFR1-3) and BLU9931 (FGFR4) on these models [89].

#### 3.4.2. Disease Modeling and Drug Mechanism Studies

Modeling the mechanism of disease progression has primarily been achievable by using traditional 2D cell lines, mouse models, and, to a limited extent, 3D tissue culture. However, all these models possess inherent limitations that compromise their predictive accuracy and their ability to recapitulate human pharmacokinetics. 3D PDTOs help bridge this gap and have been pivotal in modeling heterogeneous diseases like TNBC [15]. As briefly discussed in the introduction above, the mechanisms underlying TNBC development, resistance, and recurrence are poorly understood. Inter-tumor and intra-tumor heterogeneity present a core unresolved barrier in finding actionable targets to develop effective treatment options for patients with TNBC.

Several studies have reported using 3D PDTOs to model TNBC [52,90,91,92]. Däster et al. used patient-derived TNBC organoids derived from a BRCA2-mutated xenograft tumor and a tumor with a somatic BRCA1 mutation to model and compare their differences with wild-type TNBC tumor organoids. Their investigations identified BRCA promoter hypermethylation as a promising therapeutic marker for TNBC response to PARP inhibitors [52]. Parsyan et al. used 3D PDTOs to model TNBC and to discover the potential of CFI-400945, a novel polo-like kinase 4 (PLK4) inhibitor, to synergistically improve the efficacy of radiation therapy in TNBC [91]. Saatci et al. confirmed the utility of 3D PDTOs in modeling and identifying lysyl oxidase (LOX) inhibition as a potential strategy for inducing apoptosis in TNBC and re-sensitization to chemotherapy [90]. Sharick et al. reported the successful establishment and validation of 3D PDTOs from three individual patients with TNBC. They also developed an imaging-based assessment tool, which was validated using 3D PDTOs to predict patient outcomes [92].

In summary, these studies support that 3D PDTOs are increasingly adopted as a validated platform for gaining insights into TNBC tumorigenesis. The typical workflow for using organoids in drug screening begins with establishing a primary patient-derived organoid culture using either submerged BME culture or the ALI technique [3,10,32,34,40]. Submerged BME cultures are the most commonly used approach for drug efficacy assays and are often used to initiate organoid cultures. Once organoids form stable 3D structures, intact organoids can be treated directly with test agents and evaluated using imaging-based assays [91,92]. Alternatively, organoids can be dissociated into single cells, suspended in organoid medium and BME, and seeded into 96-well plates to generate monolayer cultures, which are commonly used for bioluminescence-based cell viability assays [52,90]. Monolayer cultures derived from 3D PDTOs are mostly intended for short-term culture and can regrow back into complex 3D structures if maintained for longer durations.

**Figure 8:**
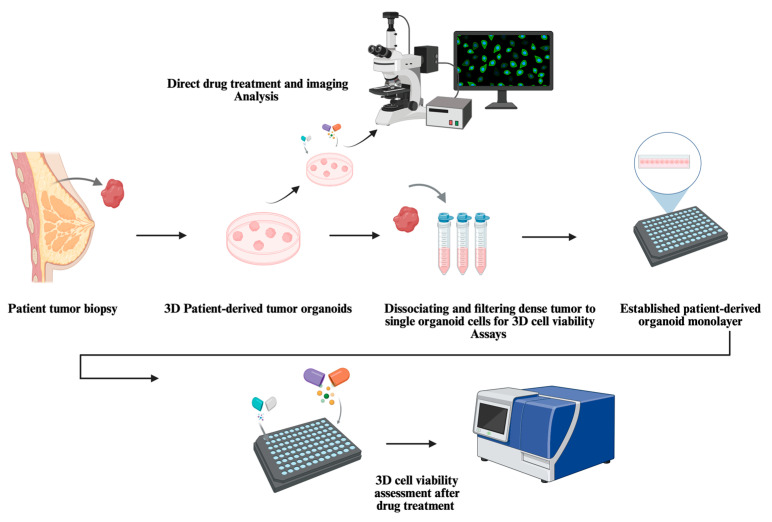
Workflow for establishing and testing 3D PDTOs in non-clinical research. Schematic overview of the experimental pipeline for PDTO-based drug response analysis: fresh tumor tissue obtained from a patient biopsy is processed to establish 3D PDTOs. Organoids are either treated directly with therapeutic compounds for imaging-based analysis or dissociated into single cells for downstream 3D viability assays. Arrows indicate the experimental sequence from biopsy to organoid establishment and assay readout. (Created in https://BioRender.com).

For disease modeling and other mechanistic studies, submerged BME or ALI culture conditions are often sufficient to maintain 3D patient-derived organoids without further dissociation. Figure 8 presents a schematic overview of a workflow for leveraging 3D PDTOs in non-clinical applications.

#### 3.4.3. Limitations of 3D PDTOs in Non-Clinical TNBC Research

One objective of this review is to identify the major barriers that must be overcome for 3D PDTO platforms to be seamlessly integrated as standard tools in routine non-clinical TNBC research. Here, we summarize the most frequently reported challenges in the literature published between 2015 and 2025. Notably, we found that most studies using 3D PDTOs in TNBC did not clearly describe the specific limitations encountered. One study that explicitly addressed these limitations was conducted by Altman et al. [16]. The authors performed single-cell transcriptional analysis of human breast cancers and model systems, including PDX and PDXO models, to compare similarities and differences in subtype proportions across models. They identified differences in metabolic transcriptional profiles among PDXOs, PDX tumors, and their corresponding patient tumors, suggesting that 3D PDTOs may have limitations in accurately modeling tumor metabolomics in TNBC [16]. More broadly, commonly cited technical challenges associated with 3D PDTO culture include hypoxia, lack of vascularization, low organoid-forming efficiency, difficulties in dose determination, limited microscopic image clarity, particularly in dense organoids, issues with reproducibility, cumbersome workflows, and high cost [14,15]. Several other studies have also briefly addressed the limitations of 3D PDTOs in TNBC research [10,15,16,93]. Table 1 highlights the outcomes of selected major TNBC studies published between 2015 and 2025 that used 3D PDTOs.

### 3.5. Applications of 3D PDTOs in Clinical TNBC Research

We identified 203 clinical studies on ClinicalTrials.gov that had registered protocols listing 3D PDTOs in their investigations. Thirty-six of the registered studies involve establishing patient-derived breast cancer organoids. The search query for TNBC organoids returned one recruiting clinical trial in the United States initiated in 2023 titled TOWARDS-II (NCT05464082). TOWARDS-II aims to conduct genomic studies and functional drug screening using patient-derived xenografts and patient-derived organoids from patients with TNBC to prospectively evaluate the correlation between PDX engraftment with recurrence [101].

Three clinical studies were identified outside the United States, one originating from Canada and two from the EU. REFLECT (NCT02732860), a recruiting clinical study sponsored by the University Health Network, Toronto, describes plans to obtain tumor specimens from participants including patients with TNBC for PDX modeling. In addition to the PDX models, the REFLECT study reports that organoid cultures from patients with TNBC may be established if sufficient fresh tissue is available. REFLECT aims to conduct a comprehensive genomic and epigenetic analysis of TNBC tumors and evaluate the utility of these human-relevant models as clinical predictors to inform personalized treatment options using chemotherapy and targeted therapies [102].

The two registered clinical studies originating from the EU featuring 3D PDTOs in their study protocols are the TRIPLEX (NCT05404321) and PDO-Neo01 (NCT07260188) clinical studies. TRIPLEX is a single-center observational study initiated by sponsors in France. TRIPLEX has enrolled approximately 163 participants and, as of December 2025, remains open for recruitment. The TRIPLEX study aims to assess the feasibility of using 3D PDTO models to evaluate their ability to discover and validate novel predictive biomarkers to determine TNBC treatment response [71].

PDO-Neo01 (NCT07260188), a clinical study initiated in Italy, aims to establish 3D PDTOs from breast cancer patients to create personalized, accurate and reliable preclinical models to gain insights into the diverse biomolecular profiles of breast cancer. The PDO-Neo01 study also describes plans to use the models to investigate the potential of biological, molecular genetic, TME, and extracellular vesicle metrics from the tumor and 3D PDTOs as biomarkers for predicting pathological complete response (pCR) to neoadjuvant chemotherapy (NAC) treatment. PDO-Neo01 highlights TNBC as a keyword in the study and as of December 2025, also remains open for recruitment [103].

Success reported from the clinical evaluation of organoids in other forms of cancers beyond TNBC supports optimism for the TOWARDS-II, REFLECT, TRIPLEX and PDO-Neo01 studies. In a phase 1/2 clinical trial by Vlachogiannis et al., 3D PDTOs from metastatic gastrointestinal (GI) tumors were shown to predict patient response to cancer treatments with 100% sensitivity, 93% specificity, 88% positive predictive value, and 100% negative predictive value [104]. Similarly, 3D PDTOs from patients with locally advanced rectal cancer (LARC) matched patient response to neoadjuvant chemoradiation (NACR) with 84.43% accuracy, 78.01% sensitivity and 91.97% specificity [105].

Our PubMed search identified a clinical case report describing the use of 3D PDTOs in this context. In that report, Ruhe et al. used functional drug testing in 3D PDTOs to support the reclassification of a cancer of unknown primary (CUP), a metastatic malignancy for which the site of origin cannot be identified at diagnosis, as TNBC [25]. Beyond clarifying the tumor’s likely origin, the organoid-based drug sensitivity assay indicated responsiveness to doxorubicin, a regimen not routinely selected for CUP. Guided by these results, the patient received doxorubicin in combination with local and regional radiotherapy and subsequently achieved a complete remission [25]. Similarly, Pan et al. reported the successful establishment of 3D PDTOs from pleural effusion-derived tumor cells from a patient with TNBC. The organoids generated from the malignant pleural effusion (MPE) continuously propagated for over 3 months, retained the triple-negative receptor expression and matched the hematoxylin and eosin (H&E) histological characteristics of the primary breast cancer and metastatic supraclavicular lymph nodes. Drug screening using the 3D PDTOs correlated to the patient’s sensitivity to capecitabine and sequencing results reported methylenetetrahydrofolate reductase (MTHFR) gene polymorphism and thymidylate synthase (TYMS) -6bp/-6bp polymorphism, which are indicative of fluorouracil effectiveness [53].

In a follow-up study, Xiao et al. leveraged matched metabolomic and genomic data from patients in the same cohort as their previous clinical study, FUTURE (NCT03805399). Their findings classified TNBC into three distinct metabolomic subgroups: C1, C2, and C3. The C1 subtype was enriched in ceramides and fatty acids, the C2 subtypes showed increased oxidation reactions and glycosyltransferase metabolites, while C3 subtypes exhibited the lowest level of metabolic dysregulation. Xiao et al. further adopted 3D PDTOs and PDX models to show that sphingosine-1-phosphate (S1P), an intermediate of the ceramide pathway, is a promising therapeutic target for LAR tumors. Although functional validation of metabolomic targets in 3D PDTOs and PDX models was similar to the effects in patients with TNBC, Xiao et al. reported that these treatments require further investigation [106].

Collectively, these studies support the feasibility of translating 3D TNBC organoid platforms for clinical decision-support applications in the future, while highlighting the need for broader validation in larger cohorts. Other cancer types in which the predictive ability of 3D PDTOs has been clinically validated for forecasting patient response include esophageal adenocarcinoma [107], gastric cancer [108], metastatic colorectal cancer [109], pancreatic cancer [110] and appendiceal cancer [111]. Regarding the limitations of 3D PDTOs in TNBC clinical research, Ruhe et al. reported that a major limitation for clinical use of 3D PDTOs is the prolonged time required to establish and expand organoid cultures before they can be used for personalized treatment selection. This timeline is further extended by the additional time needed to perform therapeutic testing. Together, these steps can substantially delay diagnostic clarification and treatment initiation, limiting real-world integration into routine care pathways. Addressing this bottleneck will require (1) optimizing culture conditions to shorten establishment and expansion times, and (2) developing standardized workflows, including clear criteria for identifying patients most likely to benefit from organoid-guided decision-making. Beyond TNBC, broader clinical applications and limitations of patient-derived organoids have been reviewed [112,113,114,115,116].

### 3.6. Landscape of Immunotherapy Research in TNBC

A primary objective of this systematic review is to evaluate the impact of immunotherapy on clinical outcomes in patients with TNBC. To clarify how advances in 3D PDTO technology have influenced immunotherapy research, we present findings from our comprehensive analysis of the role of immunotherapy in shaping the TNBC treatment landscape. We further discuss the potential of 3D PDTO models to address key barriers that currently limit the effectiveness of immunotherapeutic strategies in TNBC.

#### 3.6.1. Impact and Limitations of Immunotherapy in TNBC Treatment Landscape

In TNBC, where treatment options remain limited, the durable responses observed in subsets of patients with immune-inflamed tumors offer hope for improving outcomes. A major milestone in this setting was the FDA approval of pembrolizumab for two TNBC indications: in combination with chemotherapy for PD-L1-positive, locally recurrent unresectable or metastatic TNBC; and for high-risk early-stage TNBC in combination with neoadjuvant chemotherapy followed by adjuvant pembrolizumab after surgery [5,6]. These approvals were supported by the KEYNOTE-355 (NCT02819518) and KEYNOTE-522 (NCT03036488) trials, which demonstrated improved progression-free survival in PD-L1-positive metastatic TNBC and improved pathological complete response and event-free survival in high-risk early-stage TNBC, respectively [5,6]. Before pembrolizumab, atezolizumab, a PD-L1-blocking antibody, received accelerated FDA approval in combination with nanoparticle albumin-bound paclitaxel (nab-paclitaxel) for PD-L1-positive, unresectable locally advanced or metastatic TNBC based on the IMpassion130 (NCT02425891) phase III trial. Although this indication was later withdrawn in the United States, atezolizumab remains historically notable as the first immunotherapy approved for TNBC in the U.S. [117].

While immunotherapy has improved outcomes for selected patients with TNBC, its broader clinical impact remains limited by modest response rates and the frequent development of resistance. As briefly discussed above, both primary and acquired resistance continue to challenge the effectiveness of immunotherapy in TNBC. Previous studies have identified tumor heterogeneity, T cell exhaustion, and macrophage dysregulation within the tumor microenvironment as major factors contributing to immunotherapy resistance and treatment failure in this disease [18,118].

#### 3.6.2. Adoption of 3D PDTOs in TNBC Immunotherapy Research

We reviewed several studies published over the past decade that have leveraged 3D PDTOs to investigate the influence of intrinsic and extrinsic components of the TME on TNBC resistance. Findings from these studies demonstrate a positive correlation among specific elements of the TME, immune function, organoid viability, and the overall accuracy of 3D PDTOs in TNBC research [3,7,26,34,35].

Our query for TNBC studies using organoids for T cell exhaustion returned no published studies meeting our inclusion criteria for that category. The query to investigate the use of 3D PDTOs to model the impact of heterogeneity in TNBC immunotherapy returned only one published study. The final query for studies using 3D PDTOs to investigate TNBC-associated macrophage dysregulation, returned two eligible published results. However, several studies investigating these limitations have been published in other 3D models and traditional non-clinical research [119,120,121].

Overall, we found that 3D PDTOs remain underutilized in investigations of key factors contributing to immunotherapy resistance and failure in TNBC. 3D PDTOs accounted for a minority of reported models (11.4%), whereas most studies relied on alternative 3D systems (88.6%), including spheroids and other non-patient-derived 3D culture platforms.

### 3.7. AI Impact on 3D PDTOs in TNBC Research

The term organoid intelligence (OI) is a nascent term used to describe the convergence of AI with 3D organoids. This integration of biological sciences and *in silico* sciences enables the uncovering of new insights into the mechanism of TNBC progression. The term originates from the concept of developing brain organoids capable of computing, storing information, and executing tasks. However, the integration of AI into TNBC organoid research spans beyond OI. Our query for studies reporting the use of AI and machine learning (ML) alongside 3D PDTOs identified 2 studies.

Zou et al. developed a statistical and ML framework called Congruence Analysis and Selection of Cancer Models (CASCAM), to authenticate and select the most representative cancer models in a pathway-specific manner using transcriptomic data. They included datasets from several TNBC 3D PDTOs and confirmed the models’ congruence in predicting specific patient outcomes. The efficiency of CASCAM has yet to be validated in real-world applications [100]. Kim et al. applied ML methods for identifying novel therapeutic candidates for patients with TNBC. Their study aimed to define a consensus molecular subtype (CMS) of TNBC by integrating the expression profiles of 957 TNBC samples from published datasets. The model confirmed the recapitulation of the in-depth functional and cellular heterogeneity seen in primary and metastatic TNBC. They proposed the CMS as a relevant therapeutic prediction model that can provide insights into diagnostic markers for TNBC subtype and metastasis [94]. Other studies we found detailing the applications of AI/ML technology in TNBC research include those by Zhao et al. and Abdel-Rehim et al. [122,123].

### 3.8. Regulatory Landscape Guiding the Adoption of 3D PDTOs in TNBC Research

To gain insights into the regulatory landscape for the adoption of 3D PDTOs as human-relevant models for non-clinical and clinical research, our research examined official communication and guidance documents issued by the FDA, NIH, and EMA. Here, we present detailed insights into the major plans, policies, and standards published by regulatory authorities that have guided the adoption of 3D PDTOs over the past decade. We also provide insights into future trends in regulatory oversight.

#### 3.8.1. FDA

The FDA supports the use of human-relevant models when they are fit for purpose and appropriately address the scientific question under investigation [124]. These human-relevant models include 3D PDTOs, stem cell microphysiological systems, and computational *in silico* tools. In 2019, the FDA established alternative methods working groups to foster the development and implementation of alternative approaches to improve the efficacy of non-clinical research.

The main objective of the working group was to raise awareness and provide continuing education on the need for new human-relevant predictive models for the advancement of *in vitro*, *in vivo*, and *in silico* research. Since then, the FDA has been actively raising awareness of its concerns about the translational gap in research, especially in translational research involving immunotherapeutic options [12,125]. These concerns catalyzed the establishment of the Alternative Methods Forum Series [126]. They also published guidance documents outlining their intent to prioritize more human-relevant models in non-clinical research [127].

By 2022, the FDA formalized its agenda to prioritize alternative human-relevant models in its Focus Area of Regulatory Science (FARS) report. In this report, the FDA officially announced research prioritizing novel technologies to improve the predictive efficacy of non-clinical studies, as well as the replace, reduce, and refine (3Rs) animal alternative agenda as a key focus area. In the same year, the FDA Modernization Act 2.0 was passed. The modernization bill removed the requirement for animal studies when obtaining licenses for biosimilars and interchangeable biological products. The new act also authorized the use of certain alternatives to animal testing [128].

In April 2025, the FDA announced a groundbreaking step toward advancing public health by eliminating the need for animal testing in the development of monoclonal antibody therapies and other drugs in favor of more effective human-relevant models. The adoption of 3D PDTO and AI-based computational methods was an integral part of this initiative [129]. The authority appraised 3D organoid models as effective platforms that provide direct insights into human biological responses. They also commended the models’ ability to inform potential human toxicities that could easily go undetected in animals. This bold decision by the FDA is based on the results of extensive research efforts that the authority has been actively conducting for close to a decade.

#### 3.8.2. NIH

The NIH established a Standardized Organoid Modeling (SOM) Center in September 2025 dedicated to developing standardized organoid-based new approach methodologies (NAMs). The center aims to serve as a scientific hub for the standardization and development of reproducible, reliable, and easily accessible organoids for medicinal and biological research. The plan is to address the lack of standardized methodology for leveraging organoids in scientific research by establishing protocols tested directly on the models. The SOM center also announced plans to integrate AI, advanced robotics, imaging, and heterogeneous patient-derived samples to catalyze advancements in modern scientific research [130]. The overall goal of the NIH’s initiative is to advance the validity and accuracy of human-relevant research and curtail dependency on animal models in non-clinical research.

#### 3.8.3. EMA

Beyond the United States, we investigated trends in the adoption of 3D PDTOs in the EU. We found two key communications from the EMA that discuss the agency’s regulatory oversight and plans for implementing human-relevant research using 3D patient-derived organoids and AI to revolutionize translational research efficiency.

In 2020, the EMA released a document titled “EMA Regulatory Science to 2025,” which outlined the goal of the agency to advance patient-centered research. The document details plans to leverage the integration of science and technology to drive collaborative evidence and improve the quality of scientific evaluations. One of the key initiatives mentioned in the publication was the plans to leverage organoids and other non-clinical models to facilitate the 3Rs principles [131].

The EMA has appraised patient-derived organoids for their ability to substantially reduce the number of animal tests in select drug discovery research. In February 2025, the Agency published a NAMs document. Similar to the FDA’s concerns discussed above, the EMA emphasized the translational gap in non-clinical research. The EMA reports that approximately 80 to 90% of medications fail in clinical trials due to the poor efficiency of models used in non-clinical research [132].

**Figure 9:**
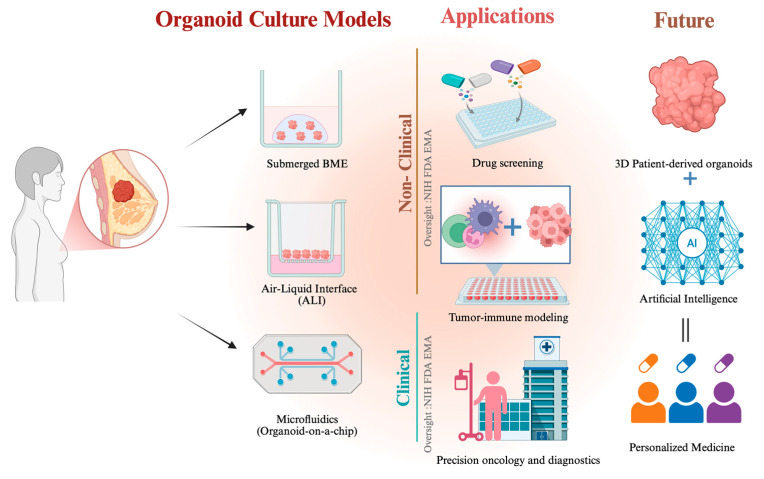
Patient-derived organoid platforms and their current and prospective applications. Tumor tissue is used to generate 3D organoids via submerged BME, ALI, or micro-organoid systems, supporting drug screening, tumor–immune modeling, and emerging precision oncology applications, with future integration of AI to enable personalized medicine. Arrows indicate the derivation of patient-derived TNBC organoids from breast tumor tissue into the three major culture platforms. (Created in https://BioRender.com).

The New Approach Methodologies publication features graphs showing the exponential growth in the adoption of NAMs in research. The adoption rate in the EU has increased exponentially from approximately 7000 publications between 2015 and 2018 to nearly 17,000 between 2021 and 2024. In the publication, the EMA highlighted the impact of organoid research on oncology. They reported that the most common applications of the 3D PDTOs were in TME and immunotherapy research. The most prominent oncology indications were breast cancer, glioblastoma, colorectal cancer, and prostate cancer [132]. Currently, the EMA and Heads of Medicines Agency (HMA) have collaborated on a work plan to facilitate the use of AI to drive scientific innovation and breakthroughs in the EU [133]. Figure 9 highlights the applications of 3D PDTOs in non-clinical and clinical research, future applications of the models, and the regulatory oversight in 3D PDTO research.

## 4. Discussion

The transformative impact of 3D PDTOs in modern research cannot be overstated. From applications in disease modeling to drug screening, these human-relevant models are catalyzing a new era of scientific research. Our systematic review is the first comprehensive assessment of the landscape of 3D PDTO adoption in TNBC research that includes regulatory insights and the impact of AI over a decade-long period. We critically analyze published studies leveraging 3D PDTOs in TNBC research from 2015 to 2025 to uncover the models, techniques, limitations and applications of 3D PDTOs used in non-clinical and clinical TNBC research.

Our analysis identified studies meeting our prespecified inclusion and exclusion criteria that used 3D PDTOs in TNBC research. The studies we analyzed were those published between 2015 and 2025, as indexed in PubMed and ClinicalTrials.gov databases. The most common application of 3D PDTOs was screening novel drug compounds and validating drug responses, and the most common model was submerged BME culture. The second most common application was their use to validate the mechanism of action of novel therapies and drug targets. These mechanistic applications showed a peak in the 2015 to 2025 organoid decade. Other applications of 3D PDTOs include discovering new combination strategies for TNBC and developing new technology to facilitate the analysis of data from 3D PDTOs.

From 2015 to 2025, several non-clinical TNBC immunotherapy studies used organoid models to investigate tumor–immune interactions and evaluate emerging immunotherapeutic strategies. However, our search showed that these models remain underutilized for studying major bottlenecks associated with immunotherapy failure, including inter- and intra-tumoral heterogeneity, T cell exhaustion, and macrophage dysregulation. Thus, 3D PDTOs represent a valuable platform for addressing important knowledge gaps in TNBC immunotherapy research.

In clinical applications for predicting immunotherapy response, homologous co-culture systems with patient-derived immune cells may be used to evaluate how a given treatment influences tumor–immune interactions [134]. Comparative flow cytometric profiling of immune cells from the original patient tumor and those retained within the organoid tumor microenvironment (TME) may further provide insight into how the TME shapes immune characteristics [135]. Likewise, comparisons between patient blood samples and 3D PDTO models may help elucidate how the tumor modulates immune components within the TME. The practical utility of these approaches, however, depends on tumor sample availability, the level of TIL infiltration, and the extent to which immune cells are maintained in organoid culture. In non-clinical research, heterologous co-cultures from human immune cells isolated from donor blood samples can be employed for studies investigating tumor–immune interactions [136,137]. In the absence of donor blood samples, specific immune cells of interest may be differentiated from immune cell lines or cloned by somatic cell reprogramming [138].

Compared with non-clinical applications, clinical adoption of 3D PDTOs remains limited. We identified only a single ongoing clinical trial in the United States and three studies outside the United States in the ClinicalTrials.gov registry employing 3D PDTOs for TNBC, and their results are yet to be determined. The case report by Ruhe et al. detailed how 3D PDTOs facilitated the identification of an unknown case as TNBC [25]. This successful clinical application of the model highlights the remarkable efficiency of the model in predicting patient treatment outcomes. Although the report did not detail the exact methodology adopted for the classification, the group mentions that the organoids predicted the patient’s response to a therapy primarily indicated for TNBC and the patient achieved complete remission following the treatment.

Collectively, these findings show that 3D PDTOs have emerged as a pivotal tool in TNBC research. However, the slower adoption rates in leveraging the models in addressing more complex non-clinical studies and clinical applications emphasize the need for more research to inform ways to broaden the application of the models and maximize their impact in TNBC research.

Facilities in the United States offering organoid-based testing for personalized oncology include the ChristianaCare’s Cawley Center for Translational Cancer Research. The institute announced the launch of an organoid core in November 2025 to cultivate 3D PDTOs to help physicians identify therapies that may be effective for individual patients [139]. Wake Forest University School of Medicine has an organoid pathology lab dedicated to establishing 3D PDTOs for personalized oncology [140]. The institute houses an organoid research center known as the Wake Forest Organoid Research Center (WFORCE) dedicated to translational research [141]. Cure First, a nonprofit facility, offers personalized testing of 3D PDTOs against an optimized panel of up to 47 anti-cancer agents including targeted therapies and antibody-drug conjugates. This personalized 3D PDTO test offered by the facility is also known as the PARIS test. The PARIS test is a Clinical Laboratory Improvement Amendments (CLIA)-certified assay based on 3D PDTO and patient genomics and has been reported to have over 90% actionability. The result turnaround time of the PARIS test is 3 weeks and patient-facing examples from the PARIS test further illustrate the potential real-world applicability of 3D PDTOs in precision oncology [142].

**Figure 10:**
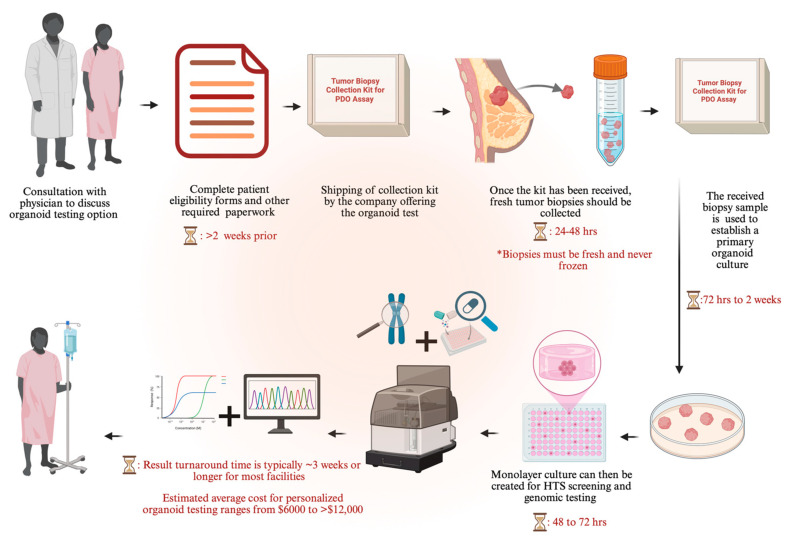
Typical workflow, timing, and cost considerations for clinical 3D PDTO testing. Black arrows indicate the sequential workflow, and circled numbers denote the major steps from physician consultation and eligibility assessment to biopsy collection, organoid establishment, assay preparation, drug/genomic testing, and result reporting. Hourglass icons indicate estimated time requirements, including >2 weeks for eligibility processing, 24–48 h for fresh biopsy collection/handling, 72 h to 2 weeks for organoid establishment, 48–72 h for monolayer assay preparation, and approximately ≥3 weeks for result turnaround. The dollar icon indicates an estimated testing cost of approximately $6000 to >$12,000. The asterisk indicates that biopsy samples should be fresh and never frozen. Timing and cost may vary depending on facility, shipping logistics, tumor type, organoid growth rate, assay complexity, and laboratory workflow. (Created in https://BioRender.com).

Outside the United States, facilities offering personalized 3D PDTO testing include Oncoforma, in Canada, BUDcare/ACCURATE in China, and Invitrocue in Germany and Hong Kong. Figure 10 shows the common workflow, cost, and timeline for clinical 3D PDTO testing seen across current facilities offering personalized organoid testing for cancer patients. Commercial facilities offering 3D PDTO testing for drug development include Xilis and HUB Organoids. Xilis utilizes a microfluidics system with patient-derived microOrganSpheres for drug efficacy testing and de-risking potential clinical candidates [143]. HUB Organoids leverages 3D PDTOs to offer drug screening, de-risking and “clinical trials in a dish” services [144].

We infer that factors contributing to the poorer adoption rates of 3D PDTOs for more advanced applications in mainstream TNBC research may be due to the cumbersome workflow, high costs, and overall maturation state of the technology. Beyond 3D PDTOs, there is a collective increase in leveraging 3D models in oncology. The higher rates of adopting less complex 3D models in TNBC research from 2015 to 2025 support our inference. 3D models such as spheroids, induced pluripotent stem cells, and embryonic stem cell-derived cancer organoids (iPSC/ESC-derived organoids) have all been employed in more complex studies in TNBC research [15,136,137]. Spheroids, defined as simple aggregates of cells that form 3D structures, are limited in their ability to recapitulate tumor intrinsic and extrinsic dynamics. Spheroids and organoids derived from human-induced pluripotent stem cell (hiPSC) lines do not faithfully recapitulate all elements of the TME and cannot model advanced stages of tumor progression.

In research investigating tumor–immune interaction within the patient TME, 3D PDTOs surpass other 3D models in recapitulating the intrinsic and extrinsic factors contributing to disease progression and treatment resistance. This is because 3D PDTOs may retain immune and stromal cells from the original patient TME. These endogenous immune and stromal components maintain patient-specific genetic alterations and signaling dynamics that cannot be faithfully reproduced in artificial *in vitro* co-culture systems employing non-patient-derived 3D models. It is interesting to note that despite the possibility that 3D PDTOs are exposed to higher drug concentrations than *in vivo* tumors, patterns of drug response and resistance in the models were seen to correspond to the patient response in most of the cases we found. Our study did not elucidate any specific dosing strategy for 3D PDTOs. More research is needed to determine the optimal dosing for 3D PDTOs. A combination of different organoids, also known as assembloids, can also be used to increase the complexity of the 3D PDTO model and may be used to model tumor metastasis.

Based on the evidence collated from this systematic review, we present key limitations that must be addressed to mitigate the risks associated with integrating 3D PDTOs into oncological research. Evidence on the disproportionate adoption of 3D PDTOs for drug response-focused application suggests the possibility of underlying barriers limiting broader application of the models in TNBC research. This higher frequency of drug-response-focused studies may also explain the limited results we found in our search to identify challenges associated with adopting 3D PDTOs in TNBC research. As such, complementary applications may be less susceptible to advanced technical challenges except for the generally known limitations like the cumbersome workflows and reproducibility challenges. The limited evidence of challenges could also reflect possible underreporting of failure rates, as studies that attempted to leverage the models without success may opt to eliminate them, leaving such instances unknown. Similar trends of possible underreporting are seen in ClinicalTrials.gov, where several protocols listing the adoption of patient-derived organoids had studies terminated or no results posted, making it difficult to assess the true extent of challenges encountered with adopting 3D PDTOs in TNBC research within the 2015 to 2025 organoid decade. It is noteworthy that the evidence collated is limited to the scope of the study detailed in the aforementioned methodology.

Another limitation that researchers using 3D PDTOs must consider is the need to clearly define the research question before determining whether 3D PDTOs are the most appropriate platform to address it. In conventional non-clinical research, the routine use of 3D PDTOs may substantially increase overall costs compared with traditional 2D models, largely because of the numerous growth factors and specialized culture components required to mimic *in vivo* conditions. Therefore, researchers must carefully consider which model is best suited to answer a given research question and when the use of 3D PDTOs is justified. As discussed earlier, multiple 3D models are available for TNBC research, each with distinct strengths and applications. Moreover, even within the range of 3D PDTO systems, the choice of a specific organoid model should be guided by the underlying scientific objective.

Currently, to the best of our knowledge, there is no consensus on which 3D PDTO culture models are best suited to address specific research questions. Thus, the outcome of the same experimental question may vary depending on the organoid culture model. To address this challenge, further research is needed to compare experimental outcomes across methods for establishing 3D PDTOs. Another option is to leverage technology to develop robust frameworks to guide this selection. Having regulatory guidance or an internationally recognized consensus on the appropriate application of 3D models in TNBC research will significantly improve the reproducibility of results from 3D PDTOs across different laboratory environments.

Researchers should also be mindful when leveraging 3D PDTOs in oncological research that the extent to which 3D PDTOs recapitulate the originating tumor depends on the quality of the tumor biopsy [145]. While the models can faithfully recapitulate the molecular and phenotypic features of the tumor sample, it is important to note that the tumor biopsy sample does not holistically represent the entire patient’s tumor but rather a niche within it. Results from 3D PDTOs are only informative about the tumor niche collected from the biopsy. Tumor cells are commonly conserved throughout the tumor, but stromal and immune cells may differ across spatial compartments within the patient’s tumor. This variation across tumor niches has been confirmed by spatial transcriptomics and single-cell analysis [146].

To improve the degree of tumor recapitulation, larger biopsies or multiple biopsies obtained from different regions of a patient’s tumor may be preferable to single-needle biopsies. This approach may help ensure that the resulting 3D patient-derived organoids more accurately represent the diverse niches within the original tumor. Multiple biopsies may also help overcome challenges in establishing successful organoid cultures when individual tumor samples are of poor quality. However, further research is needed to determine the effects of this strategy on patient safety and to assess whether differences in biopsy methods translate into higher organoid-forming efficiency and improved predictive accuracy. Finally, to enhance the clinical translation of 3D PDTOs, patient-derived microfluidic models may be particularly well suited for clinical applications because they may be established more rapidly than other 3D PDTO culture systems when generated directly, without a prior primary organoid culture step [62].

We found that AI is beginning to gain traction in TNBC research. Data generated from 3D PDTOs, together with the genetic and transcriptomic profiles of TNBC tumors, can be used to train AI models. Although our review identified ongoing efforts to integrate AI into 3D PDTO-based TNBC research, more innovative applications of AI in this area are still needed. Further studies are required to establish appropriate evaluation metrics and to validate their use in novel AI-enabled organoid assays.

When developing AI models, especially for clinical applications, researchers should apply caution and robust risk mitigation to ensure the correct use and functioning of the models. A substantial amount of discrepancy and bias in AI-enabled products stems from the complexity of their user interfaces and human-factor usability [147,148]. Thus, it is worthwhile to ensure that not only the training data but also the overall design of such models is risk-mitigated and robust. Additionally, because AI is relatively new, it is important to conduct research to assess the real-world impact and usefulness of currently available AI tools to confirm the technology’s translational potential. When developing AI-enabled technology for clinical applications, it is advisable to adopt FDA guidelines for AI/ML-enabled devices. These guidelines outline strategies to mitigate risks associated with AI integration for research applications [149].

For TNBC applications, AI models should ideally be trained on demographically diverse, clinically representative, and biologically heterogeneous datasets to account for the substantial interpatient variability that characterizes this disease. AI-enabled technologies should also clearly communicate their intended applications, the types of data used for model development, and their known limitations. Several AI/ML tools have been developed for organoid-based applications; however, many still rely on generalized frameworks that are not optimized for specific cancer subtypes or patient populations. To improve model accuracy, especially in prospective clinical settings, it may be more effective to develop disease-specific or demographic-aware AI/ML models for diagnostic and predictive applications. This is because organoids derived from different disease states and patient populations may exhibit distinct morphological, molecular, and growth characteristics. If AI models are trained on overly homogeneous datasets, their performance may decline when applied to more heterogeneous real-world samples. This phenomenon, commonly referred to as data drift, remains a major unresolved challenge in the clinical deployment of AI across healthcare [147,150,151]. In the context of TNBC, this challenge may be even more pronounced because of the marked molecular heterogeneity of the disease and the known differences in tumor biology across patient subgroups. Accordingly, future AI-enabled organoid platforms should prioritize robust external validation, continual performance monitoring, and transparent reporting standards to improve reproducibility, generalizability, and clinical trust.

### Future Directions

Overall, the trend of 3D PDTO adoption in TNBC research during the 2015 to 2025 organoid decade indicates growing readiness for, and broader use of, these models in TNBC research. This momentum among researchers and regulatory agencies, together with advances in AI, is likely to accelerate the discovery of innovative solutions that advance TNBC research in the coming organoid decades.

Despite this progress, our systematic analysis revealed substantial inconsistencies in the definition, methodology, and application of 3D PDTO models in TNBC research. A major contributing factor appears to be the limited foundational understanding of the biological and technical properties of these platforms. Although 3D PDTOs are widely recognized as capable of recapitulating important structural and functional features of the original tumor, the extent of this recapitulation across patient samples remains insufficiently defined. Moreover, differences in tissue processing, dissociation methods, culture conditions, and organoid establishment protocols may further influence model fidelity. These sources of variability likely contribute to inconsistencies in study outcomes and limit comparability across studies.

The mechanistic basis of organoid development also remains incompletely understood. While studies in non-cancer organoid systems have shown that tissues can arise from single stem cells and develop into multicellular structures, tumor organoids may be more limited in their ability to fully reproduce the complexity of the original tumor. When organoids are described as retaining elements of the tumor microenvironment, it is often assumed that key niche interactions are preserved. However, because tumor samples are mechanically and enzymatically dissociated during organoid establishment and later reaggregate into 3D structures, it remains unclear how much of the original cell–cell and cell–matrix architecture is truly maintained. More research is therefore needed to define the early developmental processes of 3D PDTO formation and to clarify how these processes affect interpretation of downstream experimental results. Some observed organoid features may reflect reassembly artifacts rather than faithful preservation of the parent tumor.

Current adoption of 3D PDTOs also often assumes that the same endpoints, analytical methods, and interpretation standards used for conventional models can be directly applied to organoid systems. Although correlations between organoid drug responses and clinical outcomes provide encouraging support for their translational relevance, additional work is needed to determine whether these same metrics are transferable across broader research applications. Because organoid systems are highly heterogeneous across cancer types and patient subgroups, the development of dedicated repositories for 3D patient-derived organoid data would be highly valuable for improving benchmarking, reproducibility, and standardization. Such resources could also facilitate multicenter validation and more rigorous comparison of organoid performance across laboratories.

Importantly, distinct organoid morphologies and differences in spatial positioning within the 3D matrix may create variable drug and nutrient gradients, thereby confounding treatment outcomes [152,153]. Accounting for organoid location, grouping organoids by morphological similarity, and comparing baseline growth characteristics may therefore improve data interpretation and reproducibility. Mathematical modeling may also help correct for some of these confounding effects. These observations suggest that organoid-based readouts should be interpreted as context-dependent outputs rather than uniform outputs.

Future standardization efforts should therefore extend beyond organoid establishment protocols to include assay design, analytical pipelines, and interpretation frameworks across different TNBC subtypes and research applications. Novel organoid assays should be guided by disease biology, the intrinsic properties of the model, and the outcome of interest. In other words, organoid assays should be purpose-built rather than applied as generic platforms.

The growing integration of AI may further accelerate the adoption of 3D PDTOs from bench to bedside. Future efforts should focus not only on applying AI to organoid data, but also on using it to address persistent challenges in experimental standardization, workflow optimization, image-based phenotyping, and quantitative analysis. AI may be especially useful for reducing operator-dependent variability and for extracting multidimensional features that are difficult to assess using conventional approaches.

With regard to TNBC immunotherapy research, greater effort should be directed toward using 3D PDTOs to address critical gaps related to tumor heterogeneity and immune dysregulation. In particular, these models may be valuable for studying T cell exhaustion, macrophage dysregulation, and inter- and intratumoral heterogeneity, which remain major bottlenecks in immunotherapy response. Better integration of immune-relevant organoid systems could therefore substantially strengthen the translational value of 3D PDTOs in TNBC research.

The reviewed literature also suggests that successful clinical translation will depend not only on biological relevance, but also on turnaround time, accessibility, reproducibility, and cost. Although organoids have shown promise in drug response studies and emerging precision oncology applications, routine implementation remains limited by variable establishment success, labor-intensive workflows, high costs, and restricted access to adequate patient samples and matched controls. Broader access to diverse, well-characterized TNBC biobanks will be essential to improve reproducibility and ensure that organoid-based findings are relevant across heterogeneous patient populations. Expanding access to deeply annotated biobanks may be just as important as improving organoid culture methods themselves.

Finally, ethical and regulatory considerations must remain central to future progress in the field. Samples should be obtained with informed consent, with careful attention to donor safety, privacy, and equitable use of patient-derived materials. As regulatory frameworks continue to evolve, clearer guidance may emerge regarding donor protection, sample governance, and responsible integration of organoid platforms into research and clinical workflows. These considerations will become increasingly important as organoid models move closer to routine translational and regulatory applications.

In brief, we anticipate that the impact of 3D PDTOs will continue to expand in the coming years, particularly through integration with AI-driven analytical approaches and ongoing regulatory efforts to facilitate their use in non-clinical research. These developments are likely to accelerate both non-clinical and clinical applications of organoid models and may support the identification of new therapeutic targets and strategies to overcome persistent bottlenecks in TNBC research. However, the long-term success of the field will depend on achieving greater mechanistic clarity, stronger standardization, and more rigorous clinical validation.

## 5. Conclusions

Our analysis indicates that the versatility of 3D TNBC PDTOs positions them as a highly promising platform for advancing the standard of care for patients with TNBC. This review shows that 3D patient-derived tumor organoids have become an increasingly important model in TNBC research, particularly in drug response studies, mechanistic investigations, and emerging tumor–immune modeling applications. Across the studies reviewed, submerged BME culture remained the most commonly used platform, whereas ALI and microfluidic systems appear particularly promising for research questions that require improved preservation of the microenvironmental context or greater physiological control.

The reviewed literature further indicates that the principal value of TNBC organoids lies in their ability to model biological features that are poorly captured by traditional 2D systems, including aspects of tumor heterogeneity, patient-specific treatment response, and selected tumor–microenvironment interactions. At the same time, the field remains constrained by important limitations, including a lack of protocol standardization, variable retention of stromal and immune components, limited access to high-quality patient samples, and uncertainty regarding how reliably ex vivo findings translate into clinical response. These limitations suggest that the future success of the field will depend not only on continued innovation, but also on stronger harmonization of methods and more rigorous validation of model performance.

Overall, translational progress in TNBC organoid research will depend not only on broader adoption, but also on improved standardization, more robust molecular and functional validation, and clearer characterization of individual 3D PDTO models in relation to the scientific questions they are intended to address. AI and evolving regulatory frameworks may support this transition, but they should be regarded as enabling tools rather than solutions to the core biological and methodological challenges that currently limit the field. If these challenges can be addressed, 3D PDTOs may evolve from promising experimental systems into more reliable platforms for both mechanistic discovery and clinically actionable precision oncology in TNBC.

## Figures and Tables

**Table 1:** Applications and Key Outcomes of Patient-Derived TNBC Organoid Models.

Author	Model	Application	Outcome
Däster et al., [52]	Submerged BME, Monolayer	Non-clinical	Discovered BRCA promoter hypermethylation as a plausible therapeutic marker for TNBC response to PARP inhibitors.
Dornhof et al., [63]	Microfluidics	Non-clinical	Developed a microfluidic organ-on-chip matrix-based platform with biosensors for precise metabolite monitoring.
He et al., [56]	Submerged BME, Monolayer	Non-clinical	Discovered a novel AXL degrader as an efficacious potential therapy for TNBC.
Jiang et al., [86]	Submerged BME, Monolayer	Non-clinical	Uncovered Bufalin as a potential therapeutic option for TNBC.
Kim et al., [94]	Not specified	New technology	Defined a consensus molecular subtype (CMS) that recapitulated in-depth functional and cellular heterogeneity of primary and metastatic TNBC.
Maimon et al., [95]	Submerged BME, Monolayer	Non-clinical	Discovered that targeting polynucleotide kinase/phosphatase (PNKP) in combination with doxorubicin synergistically inhibits TNBC growth.
Pan et al., [53]	Submerged BME, Monolayer	Clinical	Established patient-derived organoids from malignant pleural effusion for personalized medicine.
Pellizzari et al., [96]	Not specified	Non-clinical	Discovered PLK4 as a promising target for enhancing the anticancer effects of radiotherapy in TNBC.
Randolph et al., [97]	Submerged BME, Monolayer	Non-clinical	Identified LIFR signaling as a potential therapeutic target for obesity-related TNBC.
Rao et al., [87]	Submerged BME, Monolayer	Non-clinical	Discovered novel epigenetic drugs as effective therapeutic agents for TNBC and demonstrated the value of patient-derived organoids in advancing drug discovery.
Ruhe et al., [25]	Not specified	Clinical	Supported successful identification of TNBC and accurate prediction of patient response.
Ruan et al., [58]	Submerged BME	Non-clinical	Discovered iridium(III) pyridinium-N-heterocyclic carbene complex 1 as a potential anti-tumor agent for TNBC.
Sharick et al., [92]	Not specified	Non-clinical	Developed an imaging assessment tool for 3D PDTOs.
Wu et al., [98]	Submerged BME	Non-clinical	Discovered a novel potent anticancer lead compound against TNBC.
Yang et al., [99]	Submerged BME	Non-clinical	Developed a CD44-targeted and thermoresponsive nanocarrier that effectively suppressed the growth of TNBC.
Zhao et al., [50]	ALI	Non-clinical	Discovered the combination of chidamide and enzalutamide to be a potential effective treatment option for TNBC.
Zou et al., [100]	Not specified	New technology	Developed a statistical and machine learning framework using transcriptomic data to enable selection of the most representative cancer models in a pathway-specific manner.

## Data Availability

The Excel spreadsheet containing manually retrieved and filtered studies leveraging 3D PDTOs and studies pertinent to our inclusion and exclusion criteria are provided in the Supplementary Information. No new data were created or analyzed in this study.

## Supplementary Materials

The following supporting information can be downloaded at: https://www.mdpi.com/article/10.3390/cells15100922/s1, Table S1: Databases and Search Strings; Table S2: Study Protocol; Table S3: PRISMA 2020 Abstract Checklist; Table S4: PRISMA 2020 Checklist.

## Abbreviations

The following abbreviations are used in this manuscript:

2D	two-dimensional
3D	three-dimensional
3D PDTOs	three-dimensional patient-derived tumor organoids
3Rs	Replacement, Reduction, and Refinement
anti-PD-1	anti-programmed cell death protein 1
AI	artificial intelligence
AI/ML	artificial intelligence/machine learning
ALI	air-liquid interface
AXL	AXL receptor tyrosine kinase
BL1	basal-like 1
BL2	basal-like 2
BLIA	basal-like immune-activated
BLIS	basal-like immunosuppressed
BME	basement membrane extract
BRCA	breast cancer susceptibility gene
BRCA1	breast cancer susceptibility gene 1
BRCA2	breast cancer susceptibility gene 2
CASCAM	Congruence Analysis and Selection of Cancer Models
CI	confidence interval
CMS	consensus molecular subtype(s)
CUP	cancer of unknown primary
DNA	deoxyribonucleic acid
ECM	extracellular matrix
EMA	European Medicines Agency
ER	estrogen receptor
ESC	embryonic stem cells
EU	European Union
FARS	Focus Areas of Regulatory Science
FDA	U.S. Food and Drug Administration
GI	gastrointestinal tract
HER2	human epidermal growth factor receptor 2
hiPSC	human induced pluripotent stem cells
HMA	Heads of Medicines Agencies
HR	hormone receptor
IHC	immunohistochemistry
IM	immunomodulatory
iPSC	induced pluripotent stem cells
iPSC/ESC	induced pluripotent stem cell/embryonic stem cell
JIB-04	Jumonji histone demethylase inhibitor
LAR	luminal androgen receptor
LOX	lysyl oxidase
M	mesenchymal
MES	mesenchymal
ML	machine learning
mRNA	messenger RNA
MSL	mesenchymal stem-like
MTHFR	methylenetetrahydrofolate reductase
nab-paclitaxel	nanoparticle albumin-bound paclitaxel
NAMs	new approach methodologies
NIH	National Institutes of Health
OI	organoid intelligence
PARP	poly(ADP-ribose) polymerase
pCR	pathological complete response
PD-1	programmed cell death protein 1
PD-L1	programmed death-ligand 1
PDTO(s)	patient-derived tumor organoid(s)
PDX	patient-derived xenograft
PDXO	patient-derived xenograft organoid
PFS	progression-free survival
PLK4	polo-like kinase 4
PR	progesterone receptor
PRISMA	Preferred Reporting Items for Systematic Reviews and Meta-Analyses
SOM	Standardized Organoid Modeling
TILs	tumor-infiltrating lymphocytes
TYMS	thymidylate synthase
TME	tumor microenvironment
TNBC	triple-negative breast cancer
